# Laplace Transform–Based Nonparametric Test of Exponentiality against DMRL class with preservation under the Homogeneous Poisson Shock Model and applications in survival analysis and reliability

**DOI:** 10.1371/journal.pone.0349216

**Published:** 2026-06-10

**Authors:** Eman. S. El-Atfy, Alaa M. Gadallah, Arwa M. Alsahangiti, Oluwafemi Samson Balogun, Farouq Mohammad A. Alam, Mahmoud E. Bakr

**Affiliations:** 1 Department of Mathematics, Faculty of Science for (girls), Al-Azhar University, Nasr City, Cairo, Egypt; 2 Department of Basic Sciences, Thebes Higher Institute for Engineering, Thebes Academy, Cairo, Egypt; 3 Department of Statistics and Operation Research, College of Science, King Saud University, Riyadh, Saudi Arabia; 4 Department of Computing, University of Eastern Finland, Finland; 5 Department of Statistics, Faculty of Science, King Abdulaziz University, Jeddah, Saudi Arabia; 6 Department of Basic Sciences, Giza Higher Institute for Engineering and Technology, Cairo, Egypt; Universita degli Studi di Genova, ITALY

## Abstract

This paper introduces a novel, computationally efficient nonparametric test for assessing the null hypothesis of exponentiality against alternatives belonging to the Decreasing Mean Residual Life (DMRL) class. The test statistic is developed using Laplace transform techniques in conjunction with the theory of U-statistics, ensuring asymptotic normality and scale invariance. In addition, we establish the preservation of the proposed methodology under the Homogeneous Poisson Shock Model, further extending its theoretical robustness in reliability contexts. Critical values are obtained through extensive Monte Carlo simulations under both complete and right-censored data, enhancing the method’s practical applicability. A comprehensive simulation study demonstrates that the proposed test consistently outperforms classical procedures in terms of power across a wide range of alternative distributions commonly encountered in reliability and survival analysis. The usefulness of the method is further illustrated with real datasets, including COVID-19 mortality and clinical survival data, where the test successfully detects departures from exponentiality with DMRL characteristics. By combining advanced probabilistic transforms with nonparametric inference, this work provides a rigorous and scalable framework for lifetime data analysis, adaptable to the complex censoring mechanisms prevalent in medical and engineering applications.

## Section 1: Introduction

The exponential distribution is a cornerstone of reliability theory and survival analysis, primarily due to its memoryless property, which implies a constant mean residual life (MRL). Despite its mathematical simplicity, the exponential model often fails to capture the aging or wear-out behaviour observed in real-world systems, where the MRL decreases over time. Such processes are more accurately represented by the Decreasing Mean Residual Life (DMRL) class of distributions. Distinguishing exponentiality from DMRL class of life distributions is therefore critical in engineering, medicine, and risk management, where lifetime predictions guide maintenance schedules, treatment planning, and decision-making under uncertainty.

Classical procedures for testing exponentiality, such as those proposed by Hollander and Proschan [1] and Ahmad [2], have been widely employed. However, these methods may lack computational efficiency or sensitivity to subtle departures from exponentiality, particularly in the presence of censored data or complex lifetime structures.

To address these limitations, this paper proposes a novel nonparametric test based on Laplace transforms and the theory of U-statistics. The method ensures desirable properties such as asymptotic normality and scale invariance, while critical values are obtained via Monte Carlo simulations. Importantly, we establish that the proposed class is preserved under the Homogeneous Poisson Shock Model, reinforcing its theoretical robustness in reliability contexts. By combining reliability theory with modern computational techniques, the proposed framework provides a powerful and practical tool for lifetime data analysis, with applications demonstrated on real datasets including COVID-19 mortality and clinical survival studies.

## Section 2: Motivation for the Chosen Methodology

The proposed test builds on the strengths of Laplace transform techniques and U-statistics, which together provide a powerful framework for lifetime data analysis. The Laplace transform offers a compact representation of the lifetime distribution and is particularly sensitive to changes, making it well suited for detecting departures from exponentiality. U-statistics complement this by extracting information from all possible subsamples, ensuring efficiency, unbiasedness, and robustness even for moderate sample sizes. Their asymptotic normality further facilitates statistical inference. This combination achieves a balance of rigour, computational efficiency, and sensitivity to alternatives of interest, making the test especially effective for reliability and survival applications.

The concept of life distributions with decreasing mean residual life (DMRL) was first introduced by Bryson and Siddiqi [3] and Marshall and Proschan [4], leading to numerous nonparametric tests for exponentiality. Hollander and Proschan [1] developed one of the earliest procedures, followed by Bergman and Klefsjö [5], who proposed statistics suitable for censored data. Ahmad [2] later introduced a more powerful test, which was extended to randomly censored samples by Lim and Park [6]. Other significant contributions include Bandyopadhyay and Basu [7], Csörgő and Zitikis [8], Fagiuoli and Pellerey [9], Anis [10], Ray and Sengupta [11], and Abu-Youssef [12], all of whom advanced the theory and methodology for testing within the DMRL framework.

Numerous academics have suggested tests for exponentiality against different classes of life distributions using the Laplace transform technique. For instance, Atallah et al. [13] developed a new test for exponentiality versus (${NBU}_{mgf}$) life distribution. Mahmoud et al. [14] developed a testing exponentiality versus the new better than renewal used in Laplace transform order (NBRUL) class. El-Arishy et al. [15] developed a test for exponentiality against the renewal new better than renewal used in expectation (RNBRUE) class. El-Atfy et al. [16] focused on testing for the renewal new better than used in moment generating function order (${RNBU}_{mgf}$). Etman et al. [17] developed a test for the new better than renewal used in Laplace transform order with convexity (NBRULC) class. Abu-Youssef et al. [18] developed a test for the used better than aged in convex of order 2 (UBAC(2)). Gadallah et al. [19] tested for the new renewal better than used in Laplace transform order (NRBUL) class of life distributions and also tested for the new better than used in the increasing concave order. Bakr et al. [20] tested for the used better than aged in the moment generating function order ${UBA}_{mgf}.$ EL-Sagheer et al. [21] developed a test for the NBRUL class and Mhmoud et al. [22] for Testing exponentiality against exponential better than used in Laplace transform order. These works highlight the versatility of Laplace methods, which this study extends to testing exponentiality against DMRL alternatives.

The mean residual life (MRL) function is useful in many areas, including biometry, actuarial science and reliability. Let F be a continuous life distribution with a survival function $\overset{―}{F}(x)=\text{1}-F(x)$ and finite mean $\mu =\int_{\text{0}}^{\infty }\overset{―}{F}(x)\;dx$.

The MRL function is defined as


$$\begin{array}{c} m(x)=E\left(X-x|X\geq x\right)=\frac{\text{1}}{\overset{―}{F}(x)}\int_{x}^{\infty }\overset{―}{F}(u)du, \end{array}$$

where X is a random variable representing lifetime and *x* is the elapsed time.

If m(x) is nonincreasing in x≥0, then F is said to be a decreasing mean residual life (DMRL) distribution.

The lifetime random variable X is said to be DMRL, if and only if


$$\begin{array}{c} {\overset{―}{F}}^{\text{2}}(x)\geq f(x)V(x) \end{array}$$ (1)

Where,$V(x)=\int_{x}^{\infty }\overset{―}{F}(u)\;du$

A novel test statistic for evaluating exponentiality in relation to the Decreasing Mean Residual Life (DMRL) feature is presented in this paper. This is how the rest of the paper is structured. The preservation of the DMRL class of life distributions under homogeneous Poisson shock models is covered in the section on homogeneous Poisson shock models. The suggested testing method based on the Laplace transform order for complete data is presented in the Testing Exponentiality for Complete Data section. The Critical Values for Complete Data section includes power estimates for various life distributions, Pitman asymptotic efficiency calculations, and Monte Carlo simulations performed in Mathematica 13 to obtain critical values under the null distribution. The suggested methodology is extended to right-censored observations in the Critical Values for Censored Data section. Finally, the Real World Applications section uses several real datasets, such as COVID-19 mortality rates and clinical survival data, to illustrate the practical utility of the proposed test.

## Section 3: Homogeneous Poisson Shock Model

The Homogeneous Poisson Shock Model (HPSM) is a fundamental stochastic process widely employed in reliability engineering, risk analysis, queuing theory, and survival analysis to model the occurrence of randomly timed events, or “shocks,” affecting a system. This model characterizes scenarios where shocks arrive independently, at a constant average rate (denoted λ > 0), and satisfy the properties of orderliness (no simultaneous occurrences) and memorylessness.

Mathematically, the HPSM is defined by the Poisson counting process {N(t), t ≥ 0}, where N(t) represents the cumulative number of shocks up to time ‘t’.

The HPSM provides a mathematically tractable framework for analyzing system failure, degradation, or response under external stressors. Its assumptions of independence and a constant failure rate make it particularly suitable for modeling:

“Component failures” in reliability engineering, “Customer arrivals” in queuing systems, “Insurance claims” or operational losses in finance, “Particle decays” in physics, “Event occurrences” in telecommunications networks.

Despite its simplicity, the homogeneous Poisson model serves as a critical benchmark and foundational element for more complex non-homogeneous and compound shock processes. This paper explores [mention the specific focus of your research, e.g., its application to system reliability under cumulative shock damage, extensions to repairable systems, statistical parameter estimation, or limitations in real-world scenarios.

In statistics, homogeneous shock models describe systems or populations where external shocks affect all units in the same way. These models are especially used in time series analysis and panel data to capture the impact of macroeconomic events, policy changes or common disturbances.

Consider a device that is exposed to a sequence of shocks occurring randomly over time, modelled by a Poisson process with intensity λ, suppose further that the device has probability ${\overset{―}{P}}_{k}$ of surviving the first k shocks, where ${\overset{―}{P}}_{k}=\text{1}-P_{k},\;$ $\text{0}\leq {\overset{―}{P}}_{\text{0}}\leq {\overset{―}{P}}_{\text{1}}\leq \ldots =\text{1}$ Then the survival function of the device is given by


$$\begin{array}{c} \overset{―}{H}(x)=\sum_{k=\text{0}}^{\infty }\frac{(\lambda x{)}^{k}}{k!}e^{-\lambda x}{\overset{―}{P}}_{k},\;\overset{―}{h}(x)=\sum_{k=\text{0}}^{\infty }\lambda \frac{(\lambda x{)}^{k}}{k!}e^{-\lambda x}p_{k+\text{1}} \end{array}$$ (2)

where, $p_{k+\text{1}}\;=\;{\overset{―}{P}}_{k}\;-{\overset{―}{P}}_{k+\text{1}}$ is the probability mass function of at point k+1.

This formulation was first presented and thoroughly examined by Esary et al. [23], followed by Klefsjo [24], and has since grown to be a mainstay in the study of wear and shock processes. It has been demonstrated that this model preserves a number of ageing features, including IFR, DMRL, NBUE, and associated classes.

The following theorem applies to distributions F that contain the discrete DMRL feature and is consistent with the results of Esary et al. [23].

**Definition 1.** A discrete distribution $p_{k},k=\text{0},\text{1},...,\infty$ is said to be decreasing mean residual life if


$$\begin{array}{c} \overset{\text{2}}{\underset{k}{\overset{―}{P}}}\geq p_{k+\text{1}}\;\sum_{k=j}^{\infty }{\overset{―}{P}}_{k}. \end{array}$$

Let’s now demonstrate the subsequent theorem.

**Theorem 1:** If $\;p_{k}$ discrete DMRL implies that $\bar{H}(t)$ given by (2) is DMRL.

**Proof.** We need to show that


$$\begin{array}{c} {\overset{―}{H}}^{\text{2}}(x)\geq h(x)\;\int_{x}^{\infty }\overset{―}{H}(u)\;du \end{array}$$

Using the definition of $\begin{array}{c} H(x),\;h(x)\; \end{array}$ as in Eq. (2), then


$$\begin{array}{c} h(x)\;\int_{x}^{\infty }\overset{―}{H}(u)\;du=\sum_{k=\text{0}}^{\infty }\lambda \frac{(\lambda x{)}^{k}}{k!}e^{-\lambda x}p_{k+\text{1}}\int_{x}^{\infty }\sum_{k=\text{0}}^{\infty }\frac{(\lambda u{)}^{k}}{k!}e^{-\lambda u}{\overset{―}{P}}_{k}du \end{array}$$

$$\begin{array}{c} \;=\sum_{k=\text{0}}^{\infty }\lambda \frac{(\lambda x{)}^{k}}{k!}e^{-\lambda x}p_{k+\text{1}}\sum_{k=\text{0}}^{\infty }{\overset{―}{P}}_{k}\int_{x}^{\infty }\frac{(\lambda u{)}^{k}}{k!}e^{-\lambda u}du \end{array}$$

$$\begin{array}{c} =\sum_{k=\text{0}}^{\infty }\frac{(\lambda x{)}^{k}}{k!}e^{-\lambda x}p_{k+\text{1}}\sum_{k=\text{0}}^{\infty }{\overset{―}{P}}_{k}\sum_{j=\text{0}}^{k}\frac{(\lambda x{)}^{j}}{j!}e^{-\lambda x} \end{array}$$

$$\begin{array}{c} =\sum_{k=\text{0}}^{\infty }\frac{(\lambda x{)}^{k}}{k!}e^{-\lambda x}p_{k+\text{1}}\sum_{j=\text{0}}^{\infty }\sum_{k=j}^{\infty }{\overset{―}{P}}_{k}\frac{(\lambda x{)}^{j}}{j!}e^{-\lambda x} \end{array}$$

By using the DMRL property


$$\begin{array}{c} h(x)\;\int_{x}^{\infty }\overset{―}{H}(u)\;du\leq \sum_{k=\text{0}}^{\infty }\frac{(\lambda x{)}^{k}}{k!}e^{-\lambda x}{\overset{―}{P}}_{k}\sum_{j=\text{0}}^{\infty }\frac{(\lambda x{)}^{j}}{j!}e^{-\lambda x}{\overset{―}{P}}_{j}={\overset{―}{H}}^{\text{2}}(x). \end{array}$$

Which conclude the proof.

## Section 4: Testing Exponentially for Complete Data

The test presented here depends on the n-independent and identical samples $X_{\text{1}},\;X_{\text{2}},\;\ldots ,\;X_{n}$ which come from a population with a continuous distribution function F. Our goal in this section is to present a test statistic based on Laplace transform for testing the null hypothesis $H_{\text{0}}\;:\;F\;$ is exponential distribution versus $H_{\text{1}}\;:\;F\;$ belongs to DMRL class but not being exponential. The following result is basic to our development.

**Lemma 1.** Let F be a DMRL distribution; then,


$$\begin{array}{c} \int_{\text{0}}^{\infty }e^{-sx}{\overset{―}{F}}^{\text{2}}(x)dx\geq \int_{\text{0}}^{\infty }e^{-sx}V(x)\;f(x)\;dx \end{array}$$ (3)

A measure of departure from $H_{\text{0}}$ in favor of $H_{\text{1}}$ is


$$\begin{array}{c} \Upsilon (s)=\frac{\text{1}}{s}E\left(\text{1}-e^{-s\;min\left(x_{\text{1}},x_{\text{2}}\right)}\right)-\int_{\text{0}}^{\infty }e^{-s\;x}V(x)\;dF(x) \end{array}$$ (4)

**Proof.** Since F is DMRL, multiplying Eq. (1) by $e^{-sx}$and integrating over (0, ∞), w.r.to. x, then we get Eq. (3).

Since,


$$\begin{array}{c} E\left(\text{1}-e^{-s\;min\left(x_{\text{1}},\ldots ,\;x_{r}\right)}\right)=\int_{\text{0}}^{\infty }se^{-sx}{\overset{―}{F}}^{r}(x)dx \end{array}$$

Eq. (3) can written as,


$$\begin{array}{c} \frac{\text{1}}{s}E\left(\text{1}-e^{-s\;min\left(x_{\text{1}},x_{\text{2}}\right)}\right)\geq \int_{\text{0}}^{\infty }e^{-sx}V(x)\;dF(x) \end{array}$$

Thus, the result in Eq. (4) can follows.

Let $X_{\text{1}}$, $X_{\text{2}}$,..., $X_{n}$ be an independent and identically random sample drawn from a population with distribution function F(x). To construct a test statistic based on the Laplace transform, we replace the unknown survival function $\overset{―}{F}(x)$ by the empirical distribution ${\overset{―}{F}}_{n}(x),$ where


$$\begin{array}{c} {\overset{―}{F}}_{n}(x)=\frac{\text{1}}{n}\sum_{i=\text{1}}^{n}I\left(X_{i}>x\right),\;dF_{n}(x)=\frac{\text{1}}{n}\; \end{array}$$

$$\begin{array}{c} E\left(\text{1}-e^{-s\;min\left(x_{\text{1}},x_{\text{2}}\right)}\right)=\frac{\text{1}}{n^{\text{2}}}\sum_{i=\text{1}}^{n}\sum_{j=\text{1}}^{n}\text{1}-e^{-s\;min\left(X_{i},X_{j}\right)} \end{array}$$

$$\begin{array}{c} V(x)=\int_{x}^{\infty }\overset{―}{F}(u)\;du=E(X-x)I(X>x)=\frac{\text{1}}{n^{\text{2}}}\sum_{i=\text{1}}^{n}\sum_{j=\text{1}}^{n}\left(X_{j}-X_{i}\right)I\left(X_{j}>X_{i}\right) \end{array}$$

The empirical estimator of Υ(s)described in Equation (4) is obtained after substitution and simplification as


$$\begin{array}{c} \hat{\Upsilon }(s)=\frac{\text{1}}{n^{\text{2}}}\sum_{i=\text{1}}^{n}\sum_{j=\text{1}}^{n}\left[\frac{\text{1}}{s}\left(\text{1}-e^{-s\;min\left(X_{i},X_{j}\right)}\right)-e^{-s\;X_{i}}\left(X_{j}-X_{i}\right)I\left(X_{j}>X_{i}\right)\right].\; \end{array}$$ (5)

Note that under $H_{\text{0}}:\;\hat{\Upsilon }(s)=\text{0},$ while under $H_{\text{1}}:\;\hat{\Upsilon }(s)>\text{0}$, To ensure the scale invariant of the proposed test, we employ the following approach,


$$\begin{array}{c} {\hat{\Upsilon }}_{n}(s)=\frac{\hat{\Upsilon }(s)}{\overset{―}{X}}, \end{array}$$

which can be written as,


$$\begin{array}{c} {\hat{\Upsilon }}_{n}(s)=\frac{\text{1}}{n^{\text{2}}\overset{―}{X}}\sum_{i=\text{1}}^{n}\sum_{j=\text{1}}^{n}\psi \left(X_{i},X_{j}\right),\; \end{array}$$ (6)

where,


$$\begin{array}{c} \psi \left(X_{i},X_{j}\right)=\frac{\text{1}}{s}\left(\text{1}-e^{-s\;min\left(X_{i},X_{j}\right)}\right)-e^{-s\;X_{i}}\left(X_{j}-X_{i}\right)I\left(X_{j}>X_{i}\right),\; \end{array}$$ (7)

is the symmetric kernel of the Laplace transform test-based characterization of the DMRL class.

Because of its symmetric design, the estimator ${\hat{\Upsilon }}_{n}(s)\;$in Equation (5) can be represented as a U-statistic of degree two, which ensures unbiasedness and facilitates the derivation of its asymptotic features.

The subsequent theorem formally establishes the asymptotic normality of the proposed test statistic of ${\hat{\Upsilon }}_{n}(s)$. □

**Theorem 2.**

As $n\to \infty ,\;\sqrt{n}\left({\hat{\Upsilon }}_{n}(s)-\Upsilon (s)\right)$ converges asymptotically to normal with mean 0 and variance $\sigma^{\text{2}}$ given as in Eq. (10).

Under $H_{\text{0}}$, the variance $\sigma^{\text{2}}$ reduces to Eq. (11).

**Proof.** Using Eq. (7) then, let i=1 and j=2 then,


$$\begin{array}{c} \beta \left(X_{\text{1}}\right)=E\left(\psi \left(X_{\text{1}},X_{\text{2}}\right)|X_{\text{1}}\right)+E\left(\psi \left(X_{\text{2}},X_{\text{1}}\right)|X_{\text{1}}\right) \end{array}$$

Where,


$$\begin{array}{cc} E\left(\psi \left(X_{\text{1}},X_{\text{2}}\right)|X_{\text{1}}\right) & =\frac{\text{1}}{s}E\left[\left(\text{1}-e^{-s\;min\left(X_{\text{1}},X_{\text{2}}\right)}\right)|X_{\text{1}}\right]-\int_{\text{0}}^{\infty }e^{-\left(sX_{\text{1}}+X_{\text{2}}\right)}\left(X_{\text{2}}-X_{\text{1}}\right)I\left(X_{\text{2}}>X_{\text{1}}\right)dX_{\text{2}}\\ & =\int_{\text{0}}^{X_{\text{1}}}e^{-sx}\overset{―}{F}(x)dx-\int_{X_{\text{1}}}^{\infty }e^{-\left(sX_{\text{1}}+X_{\text{2}}\right)}\left(X_{\text{2}}-X_{\text{1}}\right)dX_{\text{2}} \end{array}$$

$$\begin{array}{c} \;=\int_{\text{0}}^{X_{\text{1}}}e^{-sx}\overset{―}{F}(x)dx-e^{-(\text{1}+s)X_{\text{1}}}\; \end{array}$$ (8)

$$\begin{array}{cc} E\left(\psi \left(X_{\text{2}},X_{\text{1}}\right)|X_{\text{1}}\right) & =\frac{\text{1}}{s}E\left[\left(\text{1}-e^{-s\;min\left(X_{\text{2}},X_{\text{1}}\right)}\right)|X_{\text{1}}\right]-\int_{\text{0}}^{\infty }e^{-(\text{1}+s)X_{\text{2}}}\left(X_{\text{1}}-X_{\text{2}}\right)I\left(X_{\text{1}}>X_{\text{2}}\right)dX_{\text{2}}\\ & =\int_{\text{0}}^{X_{\text{1}}}e^{-sx}\overset{―}{F}(x)dx-\int_{\text{0}}^{X_{\text{1}}}e^{-(\text{1}+s)X_{\text{2}}}\left(X_{\text{1}}-X_{\text{2}}\right)dX_{\text{2}}\\ & =\int_{\text{0}}^{X_{\text{1}}}e^{-sx}\overset{―}{F}(x)dx+\frac{\text{1}}{(\text{1}+s{)}^{\text{2}}}\left(\text{1}-e^{-(\text{1}+s)X_{\text{1}}}\right) \end{array}$$

$$\begin{array}{c} \;-\frac{\text{1}}{\left(\text{1}+s\right)}X_{\text{1}}.\; \end{array}$$ (9)

From Eq. (8), (9) then


$$\begin{array}{c} \beta \left(X_{\text{1}}\right)=\text{2}\int_{\text{0}}^{X_{\text{1}}}e^{-sx}\overset{―}{F}(x)dx+\frac{\text{1}}{(\text{1}+s{)}^{\text{2}}}\left(\text{1}-e^{-(\text{1}+s)X_{\text{1}}}\right)-e^{-(\text{1}+s)X_{\text{1}}}-\frac{\text{1}}{\left(\text{1}+s\right)}X_{\text{1}}. \end{array}$$

Then the variance is


$$\begin{array}{c} \sigma^{\text{2}}=Var\left[\beta \left(X_{\text{1}}\right)\right].\; \end{array}$$ (10)

Under $H_{\text{0}}$


$$\begin{array}{c} \beta \left(X_{\text{1}}\right)=\frac{\text{2}s+\text{3}}{(\text{1}+s{)}^{\text{2}}}-\frac{\text{1}}{\left(\text{1}+s\right)}X_{\text{1}}-{\left(\frac{\text{2}+s}{\text{1}+s}\right)}^{\text{2}}e^{-(\text{1}+s)X_{\text{1}}} \end{array}$$

Then,


$$\begin{array}{c} {\;\mu }_{\text{0}}=E\left(\beta \left(X_{\text{1}}\right)\right) \end{array}$$

$$\begin{array}{c} =\int_{\text{0}}^{\infty }\left[\frac{\text{2}s+\text{3}}{(\text{1}+s{)}^{\text{2}}}-\frac{\text{1}}{\left(\text{1}+s\right)}X_{\text{1}}-{\left(\frac{\text{2}+s}{\text{1}+s}\right)}^{\text{2}}e^{-(\text{1}+s)X_{\text{1}}}\right]e^{-X_{\text{1}}}dX_{\text{1}}=\text{0}, \end{array}$$

and the variance $\overset{\text{2}}{\underset{\text{0}}{\sigma }}$ reduces to


$$\begin{array}{c} \overset{\text{2}}{\underset{\text{0}}{\sigma }}=\frac{\text{1}}{\text{2}s+\text{3}}.\; \end{array}$$ (11)

At,

**Table:** 

S=0.5	S=0.2	S=0.1	S=1	S=2	S=5
$\overset{2}{\underset{0}{\sigma }}=0.25$	$\overset{2}{\underset{0}{\sigma }}=0.29$	$\overset{2}{\underset{0}{\sigma }}=0.31$	$\overset{2}{\underset{0}{\sigma }}=0.2$	$\overset{2}{\underset{0}{\sigma }}=0.14$	$\overset{2}{\underset{0}{\sigma }}=0.08$

The table shows that as the value of S increases, the corresponding value of $\overset{\text{2}}{\underset{\text{0}}{\sigma }}$ decreases noticeably. This trend indicates an inverse relationship between S and $\overset{\text{2}}{\underset{\text{0}}{\sigma }}$, where increasing S leads to a reduction in the initial variance. It is also observed that the change in $\overset{\text{2}}{\underset{\text{0}}{\sigma }}$ is more pronounced at lower values of S compared to higher ones.

## Section 5: Critical Values for complete data

The Monte Carlo method is a computational technique that relies on repeated random sampling and probabilistic modelling to obtain numerical solutions for problems that may be deterministic or stochastic in nature. Originating from work in the 1940s during nuclear research at Los Alamos, this method enables the approximation of complex mathematical problems that are otherwise difficult or impossible to solve analytically or through conventional numerical methods.

At its core, the Monte Carlo method involves defining a domain of possible inputs, generating random samples according to specified probability distributions, performing deterministic computations on these samples, and aggregating the results to estimate desired quantities. This approach is widely applied in various fields such as physics, chemistry, finance, engineering, and statistics, addressing tasks including numerical integration, optimization, and risk assessment.

The strength of the Monte Carlo method lies in its flexibility and scalability, especially for high-dimensional problems or systems with inherent uncertainty. However, its accuracy depends on the quality of random sampling and the number of iterations, with computational cost increasing as precision requirements grow.

Overall, the Monte Carlo method has become an indispensable tool in scientific computing, offering approximate but powerful solutions to complex problems across diverse disciplines.

**Fig 1:**
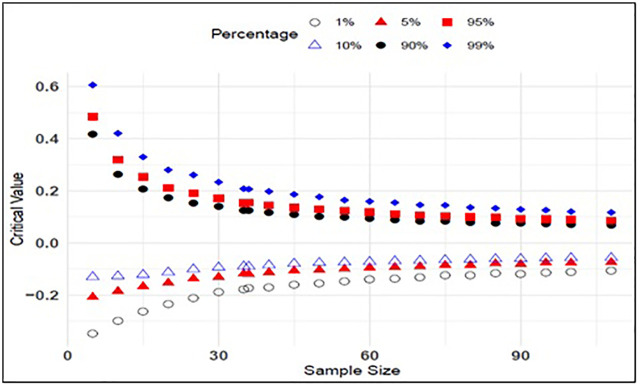
The relationship between n and C.V. at 𝐬=0.5.

**Fig 2:**
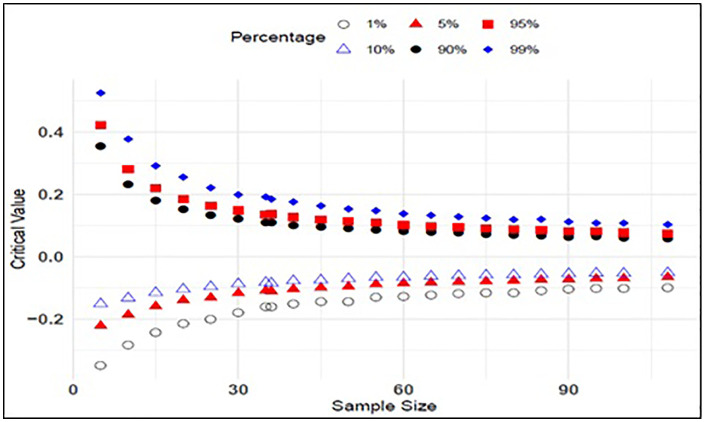
The relationship between n and C.V. at 𝐬=1.

**Fig 3:**
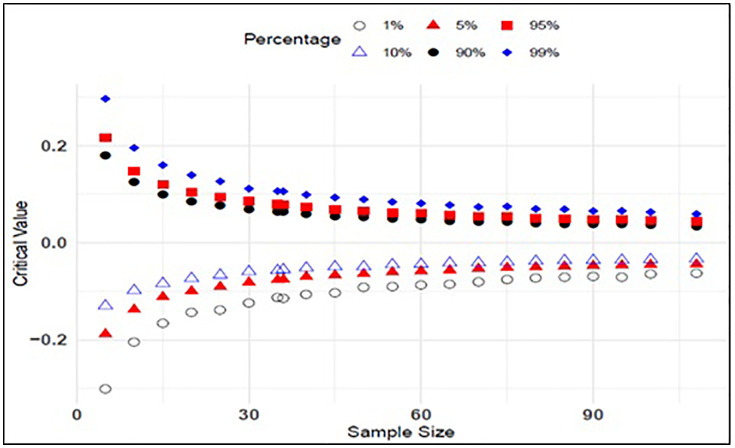
The relationship between n and C.V. at 𝐬=5.

This section computes the upper and lower percentiles of ${\hat{\Upsilon }}_{n}(s)$ in Eq. (6) utilizing 10,000 replications with sample sizes n=5(5)100,(36)(108) . These percentile points are displayed in Tables 1–3 as well as Figs 1–3. at s=0.5,  s=1 and s=5.

**Table 1:** Critical values of statistic ${\hat{\Upsilon }}_{n}(s)$ by Monte Carlo at s=0.5.

n	0.01	0.05	0.1	0.90	0.95	0.99
5	0.345974-	0.207455-	0.130346-	0.417201	0.485029	0.606619
10	0.297707-	0.184676-	0.127445-	0.263978	0.319626	0.421140
15	0.263052-	0.166844-	0.122102-	0.206985	0.253642	0.330086
20	0.233227-	0.152552-	0.112500-	0.173631	0.212488	0.280555
25	0.210472-	0.136886-	0.100778-	0.154065	0.191552	0.261095
30	0.188820-	0.130704-	0.093730-	0.140328	0.172152	0.233832
35	0.177544-	0.118095-	0.089649-	0.124575	0.153602	0.208451
36	0.171824-	0.119689-	0.090425-	0.124955	0.154764	0.206669
40	0.170610-	0.112725-	0.084874-	0.116992	0.145190	0.198080
45	0.158539-	0.106219-	0.079027-	0.109913	0.135752	0.186628
50	0.153231-	0.103351-	0.075895-	0.102017	0.129364	0.177287
55	0.145919-	0.099739-	0.074348-	0.100216	0.123668	0.164636
60	0.140351-	0.094839-	0.072194-	0.094205	0.118200	0.159865
65	0.135758-	0.092518-	0.069487-	0.088749	0.112296	0.155227
70	0.131200-	0.090002-	0.067434-	0.085105	0.106064	0.146095
75	0.123405-	0.084971-	0.065089-	0.083419	0.104234	0.144770
80	0.124346-	0.085307-	0.063277-	0.078851	0.099879	0.136373
85	0.116487-	0.078762-	0.061461-	0.076122	0.097859	0.133499
90	0.118259-	0.081513-	0.060915-	0.075638	0.093519	0.129070
95	0.113163-	0.075424-	0.058194-	0.074072	0.092077	0.125939
100	0.110220-	0.076259-	0.057340-	0.070768	0.089926	0.120462
108	0.105651-	0.073734-	0.056939-	0.068717	0.086508	0.117364

**Table 2:** Critical values of statistic ${\hat{\Upsilon }}_{n}(s)$ by Monte Carlo at s=1.

n	0.01	0.05	0.1	0.90	0.95	0.99
5	0.346671-	0.220580-	0.150723-	0.354577	0.422287	0.525202
10	0.281305-	0.185357-	0.132245-	0.233236	0.281451	0.377377
15	0.242749-	0.158145-	0.114781-	0.180681	0.220350	0.291767
20	0.213866-	0.138487-	0.103613-	0.152437	0.185509	0.255614
25	0.200648-	0.130377-	0.095713-	0.133222	0.163857	0.221437
30	0.178624-	0.116358-	0.086554-	0.121648	0.148161	0.199197
35	0.159968-	0.108255-	0.082117-	0.110436	0.136285	0.191899
36	0.160028-	0.110349-	0.084743-	0.110785	0.136947	0.185250
40	0.149339-	0.103177-	0.076711-	0.101981	0.127372	0.176250
45	0.143608-	0.098644-	0.074408-	0.096559	0.119426	0.163556
50	0.142508-	0.095178-	0.070251-	0.092181	0.113974	0.153530
55	0.128250-	0.087869-	0.065948-	0.087321	0.109205	0.147909
60	0.126109-	0.084688-	0.065301-	0.081918	0.101029	0.138372
65	0.122318-	0.082496-	0.062238-	0.080110	0.098415	0.133085
70	0.117633-	0.080283-	0.060405-	0.077249	0.096315	0.128751
75	0.115254-	0.078528-	0.058693-	0.072681	0.091299	0.124275
80	0.114185-	0.076149-	0.058077-	0.071341	0.088175	0.119503
85	0.108763-	0.073363-	0.056174-	0.067435	0.086293	0.120417
90	0.103869-	0.072248-	0.054016-	0.064394	0.081223	0.112450
95	0.100797-	0.070091-	0.052565-	0.065370	0.081431	0.108271
100	0.101165-	0.069278-	0.052943-	0.062138	0.078101	0.108346
108	0.098141-	0.064665-	0.050999-	0.058943	0.075157	0.103492

**Table 3:** Critical values of statistic ${\hat{\Upsilon }}_{n}(s)$ by Monte Carlo at s=5.

n	0.01	0.05	0.1	0.90	0.95	0.99
5	0.299714-	0.187801-	0.129831-	0.180802	0.216656	0.296495
10	0.203458-	0.137329-	0.098198-	0.125347	0.147765	0.195374
15	0.165816-	0.111351-	0.083572-	0.100572	0.120010	0.159786
20	0.142845-	0.099371-	0.073228-	0.085279	0.103499	0.139378
25	0.138755-	0.090486-	0.066478-	0.077661	0.094378	0.126548
30	0.123553-	0.081354-	0.059271-	0.069832	0.085479	0.111423
35	0.112184-	0.076166-	0.057202-	0.063911	0.078997	0.106482
36	0.113193-	0.075611-	0.055286-	0.064005	0.078049	0.105926
40	0.105181-	0.069764-	0.051648-	0.059252	0.073434	0.099019
45	0.102843-	0.066644-	0.049245-	0.055301	0.068712	0.093226
50	0.091741-	0.063806-	0.049062-	0.052859	0.065510	0.089283
55	0.090094-	0.060349-	0.044906-	0.050095	0.061222	0.084113
60	0.086083-	0.058406-	0.044063-	0.047677	0.060375	0.081186
65	0.084839-	0.056398-	0.041636-	0.045442	0.057599	0.077607
70	0.080249-	0.053303-	0.041117-	0.044008	0.054523	0.073752
75	0.075677-	0.051431-	0.039537-	0.043537	0.053665	0.074690
80	0.071910-	0.049937-	0.037703-	0.040739	0.051249	0.069622
85	0.069773-	0.048381-	0.036315-	0.039366	0.049462	0.068955
90	0.068993-	0.047156-	0.036016-	0.038887	0.047995	0.065409
95	0.069833-	0.046091-	0.035593-	0.038068	0.047769	0.065722
100	0.064517-	0.045259-	0.034351-	0.037532	0.046411	0.063177
108	0.062610-	0.044262-	0.033492-	0.034742	0.044076	0.059163

These tables and Figures show how the critical values of a statistic change with sample size at different values of parameter (s=0.5,1, and 5). It is found that as s increases; the critical values become more stable and converge faster with increasing sample size. Lower s values result in more variability, particularly in smaller samples, which indicate the strength of the suggested test.

### 5.1. The Pitman Asymptotic Efficiencies (PAE′s)

Pitman’s efficiency is based on the asymptotic behaviour of test statistics, particularly focusing on the ratio of their "efficacies", which are defined as the standardized rates of change in the test statistics’ expected values near the null hypothesis. Mathematically, it evaluates how quickly a test can detect small departures from the null hypothesis compared to another test, thus providing a rigorous criterion for optimality in large samples.

This measure assumes that the test statistics involved are asymptotically normal and that their variances stabilize as the sample size grows. It is widely used to compare parametric and nonparametric tests, especially when assessing the power of tests under contiguous alternatives. Despite its theoretical elegance, Pitman’s efficiency relies on certain regularity conditions and may not fully capture finite-sample performance or scenarios where asymptotic normality does not hold.

Overall, Pitman’s asymptotic efficiency remains a cornerstone in statistical theory, offering valuable insights into the relative strengths of competing tests and guiding the selection of optimal procedures in large-sample contexts.

We evaluated ${\text{PAE}}^{,}\text{s}$ and compared them with various other tests. Calculations are done using the Linear failure rate distribution, Makeham distribution, Weibull distribution, and Gamma distribution alternatives in reliability theory.

The PAE(Υ(s)) can be written as,


$$\begin{array}{c} PAE\left(\Upsilon (s)\right)=\frac{\text{1}}{\sigma_{\text{0}}}{\left|\frac{d}{d\theta }\Upsilon (s)\right|}_{\theta \to \theta_{\text{0}}} \end{array}$$

Where,


$$\begin{array}{c} \frac{d}{d\theta }\Upsilon (s)=\left[\text{2}\int_{\text{0}}^{\infty }e^{-sx}{\overset{―}{F}}_{\theta }(x){{\overset{―}{F}}_{\theta }}^{\backslash }(x)\;dx-\int_{\text{0}}^{\infty }e^{-sx}V_{\theta }(x)\;d\overset{\backslash }{\underset{\theta }{F}}(x)\;-\int_{\text{0}}^{\infty }e^{-sx}\overset{\backslash }{\underset{\theta }{V}}(x)\;dF_{\theta }(x)\;\right] \end{array}$$

After some mathematical calculations and by using MATHEMATECA 13 program we get the efficiencies of these families; see Table 4.

**Table 4:** Includes the asymptotic efficiencies of the test at various values of s.

Distribution	𝐒=0.5	𝐒=0.2	𝐒=0.1	𝐒=1	𝐒=2	𝐒=5
LFR	0.800000	0.83814	0.851835	0.745356	0.661438	0.515079
Makeham	0.285714	0.288111	0.288525	0.279508	0.264575	0.225347
Wiebull	1.221720	1.211540	1.206560	1.228290	1.222600	1.169350
Gamma	0.702868	0.684559	0.677205	0.724395	0.748212	0.763312

**Fig 4:**
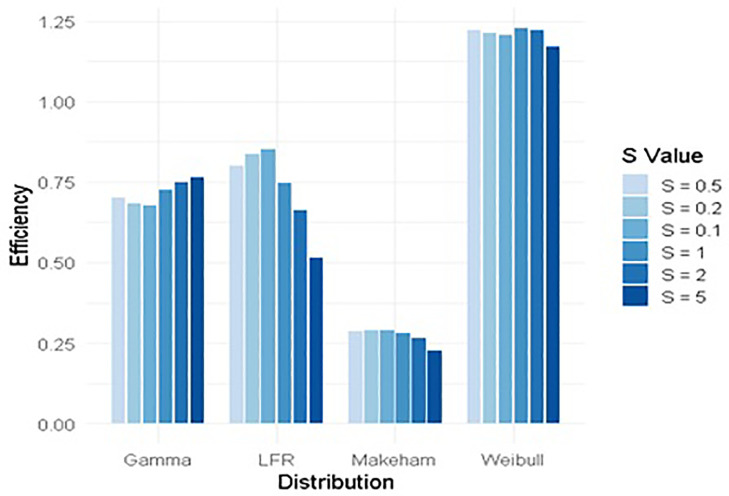
The relation between s and efficiency of Υ(s).

The asymptotic efficiencies of Υ(s) for various values of s are displayed in Table 4 and Fig 4 for the LFR, Makeham, Weibull, and Gamma distributions. Efficiency is consistently highest for the Weibull distribution and lowest for the Makeham distribution. Efficiency trends differ depending on the distribution; in contrast to the others, which typically decrease Gamma’s efficiency increases with s.

It is well established that several tests have been proposed for assessing exponentiality against the DMRL class, including those of Abu-Youssef [12], Ahmad [2], Hollander and Proschan [1]. To compare the proposed test with existing procedures targeting the same alternative, we evaluate the Pitman asymptotic relative efficiency (PARE) of the test statistic Υ(s) relative to these methods. The comparison is conducted under local alternatives converging to the null hypothesis, and the resulting PARE values, reported in Table 5, provide a quantitative measure of the asymptotic efficiency of the proposed test.

**Table 5:** Relative efficiencies for the proposed test.

Distribution		LFR	Wiebull
Υ(0.5) Υ(1) Υ(5)	Abu-Youssef [12]	0.870322 0.810875 0.560356	1.720732 1.729985 1.646972
Υ(0.5) Υ(1) Υ(5)	Ahmad [2]	0.919128 0.856347 0.591779	1.013034 1.018483 0.969610
Υ(0.5) Υ(1) Υ(5)	Hollander and Proschan [1]	0.883002 0.822688 0.568519	1.404276 1.411827 1.344080

The results in Table 5 demonstrate that the proposed test exhibits competitive, and in several cases superior, asymptotic efficiency compared to other tests, thereby confirming its effectiveness as an alternative tool for testing exponentiality against DMRL alternatives(Tables 6–8).

**Table 6:** Power estimates of the statistic ${\hat{\Upsilon }}_{n}(s)\;at\;s=0.5$.

n	θ	Distributions			
		LFR	Wiebull	Makeham	Gamma
20	2	0.4842	0.9848	0.5579	0.5394
	3	0.6022	1.0000	0.5686	0.8674
	4	0.6654	1.0000	0.5900	0.9578
30	2	0.6340	0.9994	0.7009	0.7477
	3 4	0.7417 0.8163	1.0000 1.0000	0.7236 0.7309	0.9820 0.9996
40	2	0.7582	1.0000	0.8201	0.8814
	3	0.8632	1.0000	0.8281	0.9992
	4	0.9108	1.0000	0.8341	1.0000
50	2	0.8379	1.0000	0.8848	0.9470
	3	0.9183	1.0000	0.8942	1.0000
	4	0.9497	1.0000	0.8965	1.0000

**Table 7:** Power estimates of the statistic ${\hat{\Upsilon }}_{n}(s)\;at\;s=1$.

n	θ	Distributions			
		LFR	Wiebull	Makeham	Gamma
20	2 3 4	0.5285 0.6294 0.6934	0.9857 1.0000 1.0000	0.6081 0.6405 0.6496	0.4699 0.7099 0.6458
30	2 3 4	0.6725 0.7778 0.8492	0.9993 1.0000 1.0000	0.7513 0.7737 0.7912	0.7153 0.9551 0.9847
40	2 3 4	0.7751 0.8678 0.9171	1.0000 1.0000 1.0000	0.8396 0.8604 0.8752	0.8544 0.9965 0.9998
50	2 3 4	0.8407 0.9235 0.9572	1.0000 1.0000 1.0000	0.9018 0.9148 0.9239	0.9351 0.9999 1.0000

**Table 8:** Power estimates of the statistic ${\hat{\Upsilon }}_{n}(s)\;at\;s=5$.

n	θ	Distributions			
		LFR	Wiebull	Makeham	Gamma
20	2 3 4	0.5145 0.6389 0.7266	0.9632 0.9998 1.0000	0.6851 0.7513 0.7864	0.0679 0.0004 –
30	2 3 4	0.6272 0.7612 0.8368	0.9942 1.0000 1.0000	0.7745 0.8491 0.8771	0.2513 0.0023 –
40	2 3 4	0.7242 0.8497 0.9093	0.9994 1.0000 1.0000	0.8540 0.9043 0.9263	0.5209 0.0506 –
50	2 3 4	0.7968 0.9006 0.9445	1.0000 1.0000 1.0000	0.8983 0.9402 0.9613	0.7123 0.3113 –

### 5.2. Power estimates

Statistical power is a fundamental concept in statistical analysis, representing the probability that a test will correctly reject a false null hypothesis when a true effect exists in the population. It reflects the ability of a test to avoid Type II errors, which occur when the test fails to detect an actual effect. In other words, higher statistical power indicates increased sensitivity and accuracy in identifying true differences or associations.

Estimating power prior to conducting a study is crucial for designing experiments with appropriate sample sizes, which enhances the efficiency of the analysis and reduces resource wastage. Moreover, power estimation helps minimize the risk of obtaining inconclusive or misleading results, thereby maximizing the scientific value and reliability of the research.

At significance level  α=0.05, the statistical power of the test statistic ${\hat{\Upsilon }}_{\text{n}}(\text{s})$ was estimated based on distributions including the LFR, Weibull, Makeham, and gamma. Estimates were carried out for sample sizes n=20, 30, 40 and 50 with parameter values θ= 2, 3 and 4, using 10,000 simulation replications. This approach enabled the evaluation of the test’s effectiveness in detecting true effects across different distributions and sample sizes.

The tables show that power estimates increase with large sample sizes and higher values of θ. The Weibull distribution consistently achieves the highest power; Gamma distribution power is also quite high at small values of s and becomes lowest power as s increase, while LFR fshows lower power compared to Weibull and Gamma, also Makaham distribution are generally higher than those for the LFR model but lower than Weibull distribution. Power is generally higher when s takes small values.

## Section 6: Critical Values for Censored data

Censored data refers to observations where the exact value of a measurement or event time is only partially known due to limitations in the data collection process. Instead of precise values, we have incomplete information indicating that the true value lies above, below, or within certain bounds. This type of data commonly arises in various scientific fields such as medicine, engineering, and economics.

### There are several types of censoring

Right censoring, where the event of interest has not occurred by the end of the study period, so the exact time is unknown but exceeds a known threshold. Left censoring, where the event time or measurement is known only to be less than a certain value.

Interval censoring, where the event time is known to lie within an interval but the exact time is not observed. In summary, censored data presents unique challenges but also valuable information, making it essential to apply specialized methods to extract meaningful insights in scientific research.

This research focuses on the development and application of u-tests for the EBUCL class, demonstrating their effectiveness through hypothesis testing frameworks. The study aims to provide robust statistical tools for practitioners analyzing lifetime data, enhancing the understanding of complex life behaviors beyond the classical exponential model.

Handling censored data appropriately is crucial for accurate statistical inference. Ignoring censoring can lead to biased estimates and misleading conclusions. Techniques such as survival analysis and reliability modeling have been developed to properly analyze censored observations.

The suggested test statistic employs Kaplan–Meier [25] survival function estimators where,


$$\begin{array}{c} {\overset{―}{F}}_{n}(x)=\prod_{\left(j:Z_{j}\leq X\right)}\overset{\delta (j)}{\underset{j}{C}}\;,\;X\in \left[\text{0},\;Z_{n}\right],\;and\;C_{j}=\frac{n-j}{n-j+\text{1}} \end{array}$$

Under both the null hypothesis ${\text{H}}_{\text{0}}$ and the alternative hypothesis ${\text{H}}_{\text{1}}$. The test statistic in case of censored data can be written as:


$$\begin{array}{c} \overset{c}{\underset{n}{\hat{\Upsilon }}}(s)=\sum_{i=\text{1}}^{n}e^{-s\;Z_{\left(i\right)}}\left\{{\left(\prod_{m=\text{1}}^{i-\text{1}}\overset{\delta (m)}{\underset{m}{C}}\right)}^{\text{2}}\left(Z_{\left(i\right)}-Z_{\left(i-\text{1}\right)}\right)-\left[\sum_{j=i}^{n}\left(\prod_{v=\text{1}}^{j-\text{1}}\overset{\delta (v)}{\underset{v}{C}}\right)\left(Z_{\left(j\right)}-Z_{\left(j-\text{1}\right)}\right)\right]\left(\prod_{l=\text{1}}^{i-\text{2}}\overset{\delta (l)}{\underset{l}{C}}-\prod_{l=\text{1}}^{i-\text{1}}\overset{\delta (l)}{\underset{l}{C}}\right)\right\}.\; \end{array}$$ (12)

The percentile points of our test $\overset{\text{c}}{\underset{\text{n}}{\hat{\Upsilon }}}(\text{s})$ are calculated based on 10,000 replications with samples of size n = 5(5)100, 51, 86

**Fig 5:**
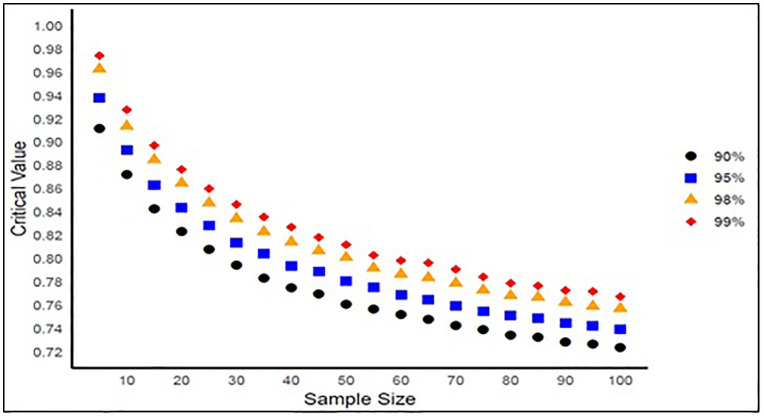
The relationship between n and C.V. at 𝐬=0.5.

**Fig 6:**
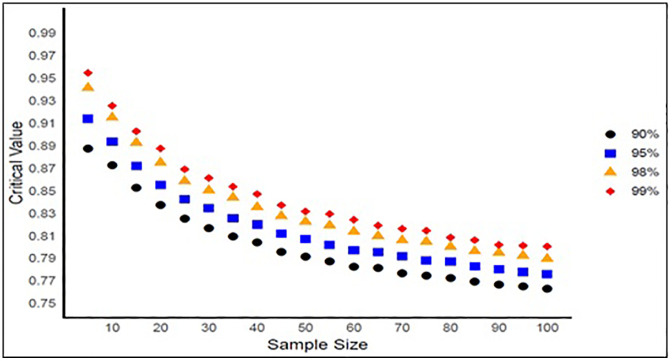
The relationship between n and C.V. at 𝐬=1.

**Fig 7:**
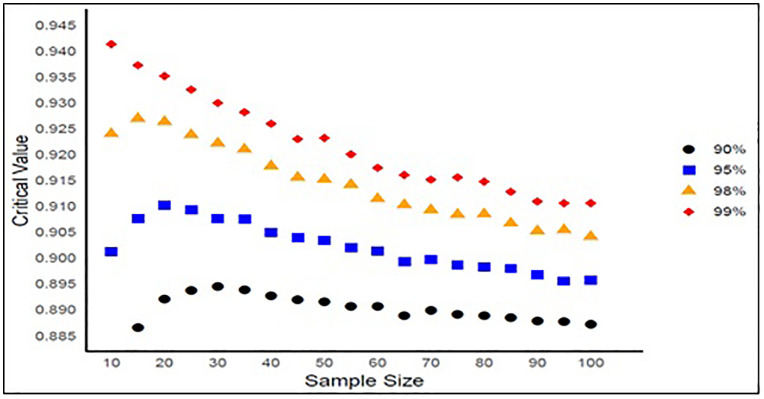
The relationship between n and C.V. at 𝐬=5.

The relationship between sample size and critical values that correspond to different significance levels is depicted in these figures. By more, clearly showing how the crucial values vary with increasing sample size, these representations successfully supplement the tabular data and improve the interpretability of the statistical patterns (Figs 5–7).

## Section 7: Real World Applications

This section analyzes real-world datasets to test the hypothesis of whether they display the exponential or DMRL properties. To examine the datasets, the proposed test statistics ${\hat{\Upsilon }}_{n}(s)$ and $\overset{c}{\underset{n}{\hat{\Upsilon }}}(s)$ mentioned in the preceding sections will be employed at 95% confidence level. The analysis employs the proposed test statistics ${\hat{\Upsilon }}_{n}(s)$ and $\overset{c}{\underset{n}{\hat{\Upsilon }}}(s)$ comparing them to critical values from Tables 1–3 and Tables 9–11 respectively. If the test statistic is under the critical value, we accept$\;H_{\text{0}}$, indicating that the data are consistent with the exponential property. However, if the test statistic above the critical value, we reject $H_{\text{0}}$ and conclude that the data have the DMRL property.

**Table 9:** Critical values of $\overset{𝐜}{\underset{𝐧}{\hat{\Upsilon }}}\left(𝐬\right)$ at 𝐬=0.5.

n	0.90	0.95	0.98	0.99
5	0.911940	0.938479	0.963034	0.974457
10	0.872058	0.893432	0.913758	0.927925
15	0.842744	0.863502	0.885024	0.897284
20	0.823449	0.843594	0.864933	0.876768
25	0.807795	0.828751	0.847797	0.860160
30	0.794757	0.813900	0.834467	0.846564
35	0.783522	0.804138	0.823125	0.835869
40	0.774738	0.793916	0.814271	0.827175
45	0.769875	0.788757	0.806873	0.818395
50	0.760815	0.780795	0.801006	0.812009
51	0.760773	0.779985	0.799050	0.809940
55	0.756563	0.775252	0.792007	0.803011
60	0.751846	0.769178	0.786645	0.798401
65	0.747616	0.764979	0.783602	0.796372
70	0.742560	0.759672	0.778928	0.790804
75	0.738827	0.754654	0.773127	0.784421
80	0.734416	0.751302	0.768274	0.778958
85	0.732833	0.749166	0.766749	0.776758
86	0.731911	0.747610	0.764234	0.777864
90	0.728326	0.744785	0.762645	0.772807
95	0.726767	0.742282	0.759121	0.771932
100	0.723859	0.739566	0.757288	0.767390

**Table 10:** Critical values of $\overset{c}{\underset{n}{\hat{\Upsilon }}}(s)$ at s=1.

n	0.90	0.95	0.98	0.99
5	0.887491	0.914136	0.941554	0.954860
10	0.872686	0.893833	0.915139	0.925627
15	0.853094	0.872166	0.892807	0.902982
20	0.837366	0.855430	0.875181	0.887677
25	0.825670	0.842603	0.858731	0.869234
30	0.816964	0.834539	0.850558	0.861543
35	0.809629	0.825890	0.844125	0.853819
40	0.804563	0.820207	0.835588	0.847228
45	0.796017	0.812019	0.827668	0.837339
50	0.791790	0.807224	0.822654	0.831806
51	0.790895	0.806803	0.822066	0.832018
55	0.787181	0.802136	0.819392	0.829581
60	0.782909	0.797295	0.813888	0.824433
65	0.781436	0.795876	0.809978	0.819262
70	0.776860	0.792045	0.806078	0.816409
75	0.774588	0.788117	0.804894	0.814709
80	0.772919	0.787159	0.800414	0.808663
85	0.769251	0.782825	0.796448	0.806311
86	0.768684	0.781579	0.796431	0.805902
90	0.766667	0.780257	0.795106	0.802118
95	0.765134	0.778020	0.792516	0.801428
100	0.762987	0.776267	0.789693	0.800726

**Table 11:** Critical values of $\overset{c}{\underset{n}{\hat{\Upsilon }}}(s)$ at s=5.

n	0.90	0.95	0.98	0.99
5	0.801256	0.852405	0.897793	0.919595
10	0.874002	0.901232	0.924026	0.941357
15	0.886601	0.907649	0.926970	0.937285
20	0.892064	0.910242	0.926363	0.935189
25	0.893797	0.909329	0.923837	0.932558
30	0.894475	0.907720	0.922197	0.929990
35	0.893901	0.907542	0.921031	0.928222
40	0.892697	0.904972	0.917811	0.925977
45	0.891893	0.903914	0.915594	0.923016
50	0.891581	0.903470	0.915232	0.923221
51	0.892023	0.903098	0.914432	0.922206
55	0.890679	0.901992	0.914214	0.920073
60	0.890605	0.901354	0.911462	0.917460
65	0.888901	0.899324	0.910289	0.916071
70	0.889837	0.899671	0.909327	0.915176
75	0.889185	0.898675	0.908430	0.915625
80	0.888905	0.898350	0.908512	0.914785
85	0.888436	0.897999	0.906743	0.912821
86	0.887813	0.897985	0.906964	0.913119
90	0.887827	0.896837	0.905248	0.910961
95	0.887740	0.895563	0.905449	0.910617
100	0.887254	0.895774	0.904154	0.910623

### 7.1. Uncensored data

**Fig 8:**
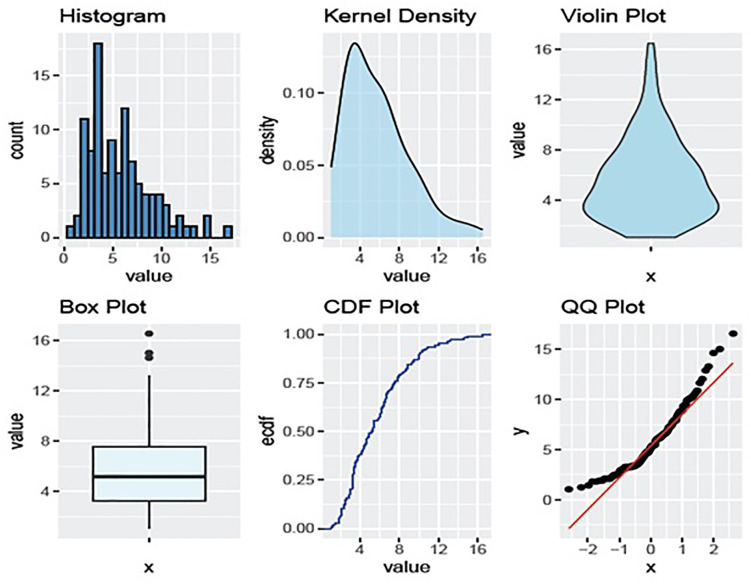
Statistical analysis for data set #1.

**Data set #1** Mexico Data holds 108 observations, demonstrating the COVID-19 mortality rates in Mexico (see Fig 8). This dataset was used by Almongy, et al. [26]. The duration of recording this data is from 4 March to 20 July 2020.

To assess the characteristics and behavior of the dataset, several nonparametric visualizations were employed, as illustrated in Fig 8. These include the histogram, kernel density plot, violin plot, box plot, cumulative distribution function (CDF) plot, and quantile-quantile (QQ) plot.

The histogram and kernel density plot reveal that the data is right-skewed and unimodal. The violin plot and box plot further support this finding by showing a longer tail on the upper side, indicating positive skewness. While most data points fall within a moderate range, a few potential outliers appear above the upper whisker in the box plot, suggesting some variability at the higher end of the distribution.

The CDF plot confirms the distribution’s skewness, as the curve rises quickly at the beginning and then flattens, implying a concentration of lower values. Finally, the QQ plot deviates from the diagonal line at the upper tail, indicating that the data does not follow a normal distribution, especially in the higher quantiles.

Thus visually validates the effectiveness of the proposed test in distinguishing between exponential and DMRL life distributions, providing practical evidence for its application in reliability and survival analysis. We computed the test statistic${\hat{\Upsilon }}_{n}(s)$ for significance level at α = 0.05. It is found that:

At s=0.5, $\;{\hat{\Upsilon }}_{n}(s)=\text{0}.\text{212209}$, this value exceeds the tabulated critical value in Table 1. This indicates that the hypothesis of exponentiality can be rejected for these data, suggesting that the dataset exhibits the DMRL property.

At s=1, ${\hat{\Upsilon }}_{n}(s)=\text{0}.\text{142548}$. Moreover, this value exceeds the critical values listed in Table 2, indicating that the data exhibit the DMRL property.

At s=5, ${\hat{\Upsilon }}_{n}(s)=\text{0}.\text{034659}$, Furthermore, this value is less than the critical values presented in Table 3, indicating that the data are consistent with the exponential distribution.

**Fig 9:**
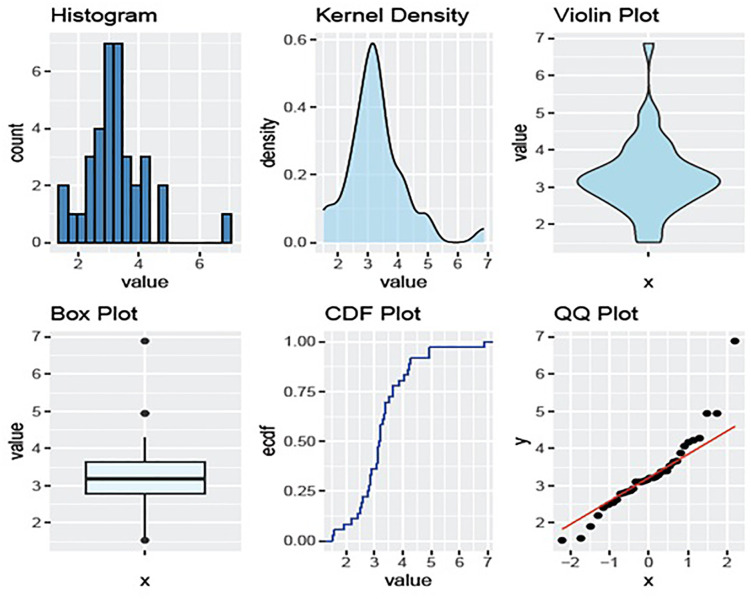
Statistical analysis for data set #2.

**Data set #2** This datasets contain 36 observations showing the mortality rates of COVID-19 patients in Canada (see Fig 9) from 10 April to 15 May 2020. This dataset was used by Almetwally et al. [27].

To assess the characteristics and distributional behavior a range of nonparametric visual tools were employed, as presented in Fig 9. The histogram and kernel density plot reveal a symmetrical and unimodal distribution centered around value 3.5, indicating a bell-shaped structure. The violin plot supports this observation, showing a balanced density on both sides of the center with no significant skewness.

The box plot shows a relatively narrow interquartile range and a few mild outliers above the upper whisker, particularly near values 5 and 6. Despite these outliers, the central mass of the data appears tightly clustered around the median. The CDF plot exhibits a smooth and steep incline near the center, confirming the concentration of values around the median.

Lastly, the QQ plot shows that most data points fall close to the reference line, with slight deviation at the upper tail, indicating that the data is approximately normally distributed but with some heavier tails. Using test statistic in Eq. (6) the results are:

At s=0.5, ${\hat{\Upsilon }}_{n}(s)=\text{0}.\text{403182}$. Moreover, this value exceeds the critical values listed in Table 1, indicating that the data exhibit the DMRL property.

At s=1, ${\hat{\Upsilon }}_{n}(s)=\text{0}.\text{266219}$. Moreover, this value exceeds the critical values listed in Table 2, indicating that the data exhibit the DMRL property.

At s=5, ${\hat{\Upsilon }}_{n}(s)=\text{0}.\text{060929}$. Furthermore, this value is less than the critical values presented in Table 3, indicating that the data are consistent with the exponential distribution.

**Fig 10:**
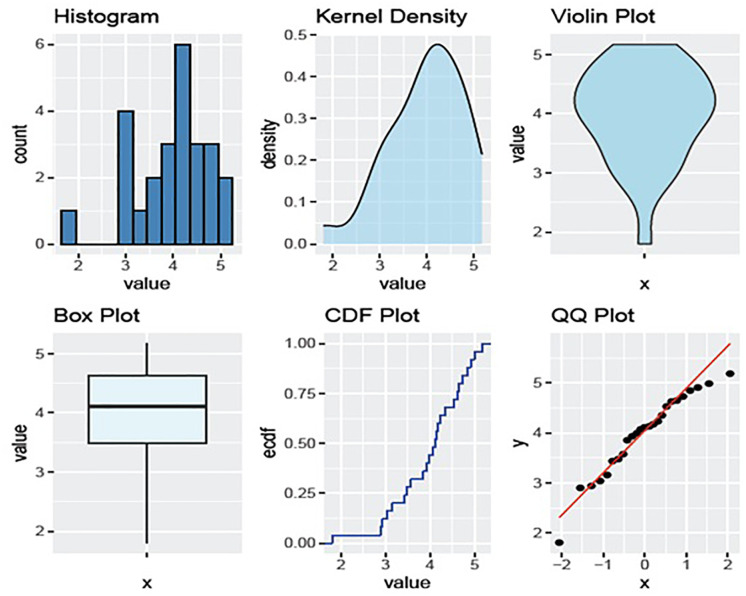
Statistical analysis for data set #3.

**Data set #3** Examine the information studied by Grubbs [28] (see Fig 10), this dataset represents the inter arrival times of 25 customers at a facility.

To assess the distributional characteristics, Fig 10 presents a suite of nonparametric visualizations, the histogram and kernel density plot reveal a right-skewed, unimodal distribution, with the majority of observations concentrated at lower values and a gradual tail extending toward higher values. This asymmetry is further highlighted in the violin plot, which shows a denser region on the left and a longer tail on the right, indicating positive skewness.

The box plot confirms the presence of skewness and displays several mild outliers above the upper whisker, reflecting a small number of unusually high values in the data. Despite these outliers, the interquartile range remains relatively compact, suggesting that most data points are clustered near the lower end of the scale.

The empirical CDF plot exhibits a steep rise at lower values, consistent with the concentration of data in this region, followed by a gradual approach to the maximum, further indicating the presence of a long right tail.

The QQ plot shows a noticeable deviation from the reference line in the upper quantiles, confirming that the data depart from normality due to heavier upper tails.

It is straightforward to demonstrate that

At s=0.5, ${\hat{\Upsilon }}_{n}(s)=\text{0}.\text{385414}$. a Moreover, this value exceeds the critical values listed in Table 1, indicating that the data exhibit the DMRL property.

At s=1, ${\hat{\Upsilon }}_{n}(s)=\text{0}.\text{234508}$. Moreover, this value exceeds the critical values listed in Table 2, indicating that the data exhibit the DMRL property.

At s=5, ${\hat{\Upsilon }}_{n}(s)=\text{0}.\text{050248}$. Furthermore, this value is less than the critical values presented in Table 3, indicating that the data are consistent with the exponential distribution.

### 7.2. Censored data

**Fig 11:**
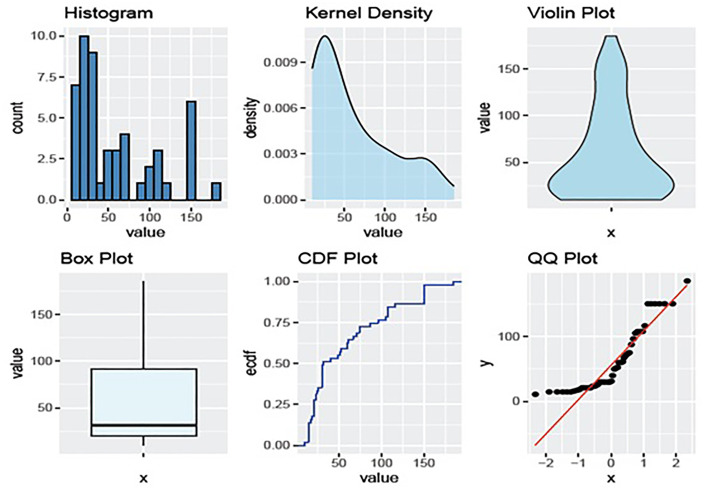
Statistical analysis for data set #4.

**Dataset #4** Consider the data in Mahmoud et al. [29]. This dataset comprises 51 liver cancer patients from the Elminia Cancer Center, as reported by the Ministry of Health in Egypt, who were admitted in 1999. Among these, 39 cases represent complete (non-censored) data, while the remaining cases are right-censored. Data visualization plots are presented in Fig 11.

To further investigate the distributional properties, Fig 11 presents a comprehensive set of nonparametric visualizations.

The histogram and kernel density plot indicate a bimodal distribution, with two distinct peaks observed around values 2 and 5. This suggests the presence of two subgroups or clusters within the data. The violin plot reinforces this observation, showing a widened density at both lower and higher values, and a narrower region in the center.

The box plot reveals a relatively wide interquartile range, reflecting the spread between the two modes. There are a few mild outliers above the upper whisker, but the majority of data points are distributed between the two main peaks. The CDF plot displays two sharp increases, further confirming the bimodal nature of the data.

The QQ plot shows substantial deviations from the reference line, particularly in the lower and upper tails, indicating that the data do not follow a normal distribution and are influenced by the presence of two modes.

The results indicate that

At s=0.5, $\overset{c}{\underset{n}{\hat{\Upsilon }}}(s)=\text{5}.\text{85046}*{\text{10}}^{-\text{42}}$. Furthermore, this value is less than the critical values presented in Table 9, indicating that the data are consistent with the exponential distribution.

At s=1, $\overset{c}{\underset{n}{\hat{\Upsilon }}}(s)=\text{6}.\text{76048}*{\text{10}}^{-\text{70}}$. Furthermore, this value is less than the critical values presented in Table 10, indicating that the data are consistent with the exponential distribution.

At s=5, $\overset{c}{\underset{n}{\hat{\Upsilon }}}(s)=\text{1}.\text{42177}*{\text{10}}^{-\text{98}}$. Furthermore, this value is less than the critical values presented in Table 11, indicating that the data are consistent with the exponential distribution.

**Fig 12:**
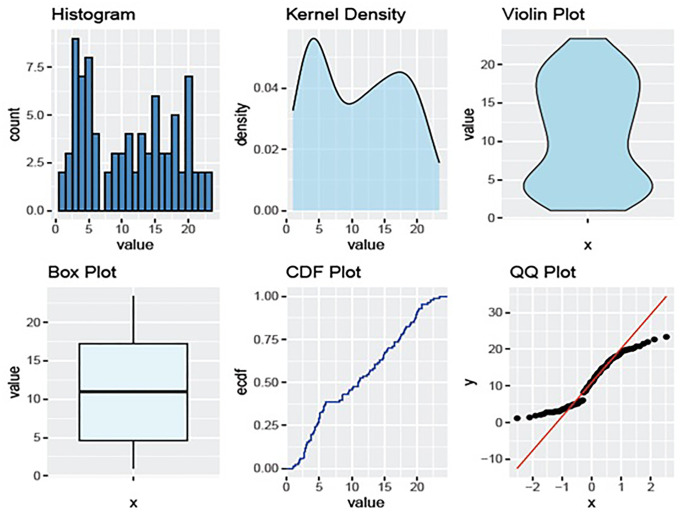
Statistical analysis for data set #5.

**Dataset #5** Based on the right-censored data for lung cancer patients reported by Peña [30] (see Fig 12), the dataset consists of 86 survival times (in months), of which 22 are right-censored. Considering the entire set of survival data both censored and non-censored and computing the test statistic as defined in Eq. (12).

To further evaluate the distributional features Fig 12 presents a comprehensive set of nonparametric visualizations such as

The histogram and kernel density plot indicate a left-skewed, unimodal distribution, with most observations concentrated at higher values and a tail extending toward lower values. The violin plot supports this observation, showing a wider density at the upper end and a tapering tail on the left, which highlights the negative skewness.

The box plot reveals a relatively tight interquartile range, but with several mild outliers below the lower whisker, reflecting a few unusually low values in the data. Despite these outliers, the majority of the data is clustered near the upper end of the scale.

The empirical CDF plot shows a gradual increase at lower values and a steeper incline at higher values, consistent with the accumulation of data points toward the upper end. The QQ plot demonstrates a clear deviation from the reference line in the lower quantiles, confirming the departure from normality due to heavier lower tails.

By using Eq. (12) it was found that

At s=0.5, $\overset{c}{\underset{n}{\hat{\Upsilon }}}(s)=\text{0}.\text{010471}$. Furthermore, this value is less than the critical values presented in Table 9, indicating that the data are consistent with the exponential distribution.

At s=1, $\overset{c}{\underset{n}{\hat{\Upsilon }}}(s)=\text{0}.\text{000177942}$. Furthermore, this value is less than the critical values presented in Table 10, indicating that the data are consistent with the exponential distribution.

At s=5, $\overset{c}{\underset{n}{\hat{\Upsilon }}}(s)=\text{9}.\text{40006}\times {\text{10}}^{-\text{14}}$. Furthermore, this value is less than the critical values presented in Table 11, indicating that the data are consistent with the exponential distribution.

## 8. Conclusions

This study develops a novel, computationally efficient test for exponentiality against the DMRL class of life distributions by combining Laplace transform techniques with U-statistics. The test exhibits desirable theoretical properties such as scale invariance and asymptotic normality, and its performance has been thoroughly validated through Monte Carlo simulations under both complete and right-censored data. The results consistently demonstrate that the proposed procedure offers superior power and sensitivity in detecting departures from exponentiality compared to classical approaches.

From a theoretical standpoint, we also examined the preservation of the DMRL property under the Homogeneous Poisson Shock Model. This result reinforces the practical relevance of the proposed methodology, as such models play an important role in reliability theory and survival analysis.

The applicability of the test was further illustrated through several real-world datasets, including COVID-19 mortality rates in Mexico and Canada, customer interarrival times, and censored medical survival data. Across these diverse applications, the test successfully distinguished between exponential and DMRL behaviors, providing empirical evidence of its robustness and practical utility. In particular, the test proved effective for both uncensored and censored data, making it well suited for contemporary survival and reliability problems where incomplete observations are common.

The contributions of this work can be summarized as (i) a novel test statistic with rigorous asymptotic theory, (ii) demonstration of DMRL preservation under the Homogeneous Poisson Shock Model, and (iii) successful application to real-world datasets, confirming both robustness and interpretability.

Future research may extend this framework to other distribution classes, multivariate survival problems, and modern machine learning–based survival techniques.

In conclusion, this study provides a rigorous, efficient, and practically relevant testing methodology that bridges classical reliability theory with modern statistical and computational tools, advancing the analysis of lifetime data in both theory and application.
